# Editorial: medical imaging modeling

**DOI:** 10.1186/s42492-019-0037-2

**Published:** 2019-12-29

**Authors:** Zhengrong Jerome Liang

**Affiliations:** Laboratory for Imaging Research and Informatics (IRIS), State University of New York, Stony Brook, New York, 11794 USA

Medical imaging can be categorized broadly into applied science or more narrowly into imaging science. As applied science, it encompasses the middle ground between basic science and engineering technology, where a definite practical end is in mind, and where the approach may lead to new discoveries in basic science as well. As imaging science, it explores the nature of visualization.

More specifically, medical imaging visualizes the inner world of the human body noninvasively. Its primary objective is to delineate the structures and map the functions of organs and tissues, based on the principles of physics, mathematics, engineering, computer science, physiology and biology.

Due to the complexities of the human body and the associated signals generation and detection, the tasks of delineating the structures and mapping the functions of the organs and tissues can be very challenging. Modeling or simplifying the complexities to visualize the major attributes of the structures and functions is a commonly adapted strategy. While all models reported in the literature are wrong in terms of describing or representing the complexities, they are very useful and have been contributing to the accumulation of current knowledges about our living body in various conditions.

This special issue calls for papers which would provide some innovative (revolutionary) ideas or models for simplifying the complexities with some preliminary demonstrations and would furthermore provide some insightful discussions about the impacts on and possible future directions of the corresponding fields. The ten papers presented in this special issue can be summarized accordingly.

Computed tomography (CT) is now a widely used imaging modality for screening and diagnosis, emergency medicine, image-guided interventions and monitoring of therapeutic responses because of its excellent capabilities of delineating the structures of the organs and tissues and mapping the contrast material dynamic distributions through the tissues [1]. However, when it is applied to cardiac and pulmonary imaging studies, the motions of heart and lungs complicate the image reconstruction task. The four-dimensional cone-beam CT (4D-CBCT) paper of Zhang, Huang and Wang provides a model of including motion estimation and machine learning to mitigate the motion complexity challenge. Other approaches to address this complexity can be found in the cited references.

For tomographic image reconstruction of a continuous region from limited number of projections around the region, sparse-view problem always exists unless the number of the projections goes to infinity [2]. The problem can be significant in some cases, depending on the applications. The work of Larry Zeng on sparse-view tomography presents a model which addresses the non-linear problem by integrating a non-linear displacement function into a simple linear interpolation. Other models for the sparse view tomography can be found in the cited references, and a more sophisticated model was recently presented for low-dose CT imaging [3].

The beauty of tomographic image reconstruction from projections is seen from the art of filtered back-projection (FBP) reconstruction, which is so far a mathematically-exact solution of the reconstruction in the absence of data noise. However, when the data counts are limited, for example for low-dose CT [4], modeling the count statistics has shown great potential to improve the reconstruction quality. A classic model for Poisson noise statistics is presented by the well-known Expectation-Maximization (EM) algorithm [5, 6]. By theory, the reconstruction problem in limited counts shall be formulated as an optimization operation, given the measured counts, leading to the Bayesian image reconstruction framework [7]. According the Bayes’ law, Bayesian medical image reconstruction remains an unsolved statistical optimization problem because the a priori probability distribution remains a challenging task and, even if an ad hoc prior model is proposed, the associate parameters are remaining as a variable. The paper of Zeng and Li on extension of EM algorithm to Bayesian algorithm provides some insights how these two algorithms are connected.

Positron emission tomography (PET) has many attractive properties as a functional imaging modality to map the tissue heterogeneity at the molecular level, thus called molecular imaging modality [8]. While the radiotracer is labeled at the molecular level of microns (μm) scale, the detection of the radiotracer decay is at the millimeter (mm) scale. Great efforts have been devoted to improve the spatial resolution of localizing the decay sites. One example is the consideration of the time-of-flight (TOF) information of the two 512 keV gamma rays of positron annihilation. The TOF information is associated with the line-of-response (LOR) measurement, and is routinely implemented in an iterative image reconstruction algorithm. The study from Zeng, Li and Huang explores an alternative implementation, where the LOR is back-projected first, followed by filtering in the back-projection domain with an analytical model.

The above four papers focus on image reconstruction, where the ultimate goal is to bring as much information as possible into the reconstructed images to achieve a desired clinical task. For medical diagnosis, imaging the tissue heterogeneity is probably the most important task, because the heterogeneity is a footprint of lesion evolution and ecology, and an indicator of lesion progress and response to intervention [9–11]. The tissue heterogeneity has been shown to be effectively characterized by tissue textures [12–14]. The tissue textures are entirely dependent on the image contrast. For CT imaging, the image contrast depends entirely on the X-ray energy and, therefore, spectral CT can alter the tissue textures for the task of tissue characterization. The paper of Gao, Shi, Cao, et al., presents a Bayesian image reconstruction of spectral CT framework with enhanced tissue textures, followed by a texture analysis along the spectral dimension for computer aided diagnosis (CADx) of colorectal polyps for colon cancer prevention. This exploratory study is probably the earliest example of focusing the tissue textures into an integrated pipeline from image reconstruction and processing to CADx. In addition, the known normal tissues of muscle, fat, bone, and lungs are incorporated into the Bayesian framework as a priori knowledge and, therefore, the tissue-specific texture prior model is no longer ad hoc prior model.

In the following five papers of this special issue, the focus is shifted from image reconstruction to image processing toward computer-aided detection (CADe) and CADx of abnormalities. As noted in refs. [10, 15], a lesion is embedded in its particular environment. Traditionally, CADx limited the attention on the lesion volume, ignoring its surrounding environment. The model presented by Zheng, Qiu, Aghael, et al. includes both the lesion volume and its surrounding environment. Furthermore, the investigators propose a machine learning strategy to extract the corresponding global features for improved prediction of cancer risk and prognosis. Other models to address the complexity of lesion itself and its surrounding environment can be found in the cited references.

Since the report of radiomics as an integrated lesion descriptor, including image features, experts’ text descriptions and lesion genetics [16], many radiomics features have been proposed. When these radiomics features are input to a machine learning classifier for lesion diagnosis, one or a few features with contributions of more or less variation (due to redundancy and other causes) would accumulate toward a significant impact to the final classification outcome. Evaluating the robustness of each radiomics feature is very necessary. The paper of Cattell, Chen, and Huang present an example of research efforts to address this important topic in the radiomics field.

As mentioned above in the paper of Gao, Shi, Cao, et al., tissue texture patterns effectively represent the tissue heterogeneity and, therefore, are considered as imaging biomarkers. Many texture descriptors (also called texture features in terms of quantitative measures or mathematical expression) are also investigated within the radiomics framework. Robustness of texture descriptors is an important research topic as shown by the work of Cattell, Chen, and Huang. The paper of Cao, Pomeroy, Gao, et al. takes the gray-level co-occurrence (GLCM) texture descriptor as an example to explore the possible causes of the variations, and provides a solution to minimize the variations by adaptive machine learning. More importantly, this paper of Cao, Pomeroy, Gao, et al. takes the GLCM as a texture image and explores the multiscale texture sampling opportunity in a similar manner as multiscale image analysis.

As mentioned above, imaging the tissue heterogeneity is probably the most important task in medical diagnosis, image-guided intervention, and treatment response quantification [9–14]. Thus, multimodal imaging becomes a desirable approach to acquire multiscale, multispectral, and multiple-source images. The paper of Ma, Lu, Wang, et al. presents another very valuable imaging modality, called hyperspectral imaging, to address the challenge of differentiating tumor and benign tissues for optimal surgical purpose.

Lung cancer remains the leading cause of cancer-related deaths. Detecting the lung nodules, the precursor of lung cancer, is the primary consideration to prevent and treat the disease early. Currently the task of detecting the nodules is performed by low-dose CT (LdCT) scanning, followed by radiologists’ interpretation of the reconstructed LdCT chest images. Artificial intelligence (AI) has been traditionally applied to the reconstructed images to mimic the experts’ interpretation. During the image reconstruction processing from acquired raw data to chest images, additional variables may be introduced, which can compromise the detection task. Therefore, a logic strategy to accomplish the detection task is taking the AI-enabled learning during the entire process from the raw data to the reconstructed images. The paper of Gao, Tan, Liang, et al. presents such a logic strategy, which has shown significant improvement over the traditional reconstructed image-focused approaches.

In summary, the ten papers in this special issue cover a wide range of modeling strategies to address the complexity in medical diagnosis, image-guided intervention, and treatment response quantification, which is usually impossible to solve without innovative modeling strategy.
